# Influence of Plant Population and Nitrogen-Fertilizer at Various Levels on Growth and Growth Efficiency of Maize

**DOI:** 10.1155/2013/193018

**Published:** 2013-09-15

**Authors:** M. I. Tajul, M. M. Alam, S. M. M. Hossain, K. Naher, M. Y. Rafii, M. A. Latif

**Affiliations:** ^1^Institute of Agriculture, Tokyo University of Agriculture and Technology, Tokyo 183-8509, Japan; ^2^Department of Crop Botany, BSMR Agricultural University, Salna, Gazipur 1701, Bangladesh; ^3^Department of Crop Science, Faculty of Agriculture, Universiti Putra Malaysia, 43400 UPM Serdang, Selangor Darul Ehsan, Malaysia; ^4^Institute of Tropical Agriculture, Universiti Putra Malaysia, 43400 UPM Serdang, Selangor Darul Ehsan, Malaysia; ^5^Bangladesh Rice Research Institute, Gazipur 1701, Bangladesh

## Abstract

Field experiments were conducted to evaluate plant population and N-fertilizer effects on yield and yield components of maize (*Zea mays* L.). Three levels of plant populations (53000, 66000, and 800000 plants ha^−1^ corresponding to spacings of 75 × 25, 60 × 25, and 50 × 25 cm) and 4 doses of N (100, 140, 180, and 220 kg ha^−1^) were the treatment variables. Results revealed that plant growth, light interception (LI), yield attributes, and grain yield varied significantly due to the variations in population density and N-rates. Crop growth rate (CGR) was the highest with the population of 80,000 ha^−1^ receiving 220 kg N ha^−1^, while relative growth rate (RGR) showed an opposite trend of CGR. Light absorption was maximum when most of densely populated plant received the highest amount of N (220 kg N ha^−1^). Response of soil-plant-analysis development (SPAD) value as well as N-content to N-rates was found significant. Plant height was the maximum at the lowest plant density with the highest amount of N. Plants that received 180 kg N ha^−1^ with 80,000 plants ha^−1^ had larger foliage, greater SPAD value, and higher amount of grains cob^−1^ that contributed to the maximum yield (5.03 t ha^−1^) and the maximum harvest index (HI) compared to the plants in other treatments.

## 1. Introduction

 Maize (*Zea mays* L.) is the third most important grain crop in the world after wheat and rice [1] and has a great utility in agroindustry. It is also of interest to report that calorie yield in maize is two and halftimes higher than that in paddy-rice [2]. In Bangladesh, the cultivation of maize has been gaining popularity in recent years. It is now becoming an important cereal crop for its high productivity and diversified use. The agroclimatic condition of Bangladesh is favorable for its cultivation all year round. However, the average yield of local maize in the country is comparatively low (1.06 t ha^−1^), whereas the newly released varieties have the potential to produce more than 5.2 t ha^−1^ [3, 4]. Agronomic practices such as seed rate, plant population and fertilizer management are known to affect crop environment, which influence the growth and ultimately the yield [5]. Optimum population nitrogen (N) levels should be maintained to exploit maximum natural resources, such as nutrients, sunlight, and soil moisture, to ensure satisfactory growth and yield. High density is undesirable because it encourages inter plants competition for resources.

 N has been found to be the most important nutrient for maize production [6]. Biomass production of a crop largely depends on the function of leaf area development and consequential photosynthetic activity [7]. Photosynthetic rate can substantially be increased with N-fertilization. Application of N-fertilizer has also been reported to have significant effect on grain yield and quality of maize [8]. Hardas and Karagianne-Hrestou [9] reported that 180 kg N ha^−1^ was optimum for maize, while Singh et al. [10] observed that application of 200 kg N ha^−1^ increased grain yield of maize. However, a substantial percentage of applied N is also lost due to volatilization, leaching, and denitrification. Therefore, N should be applied in such a way that would maximize its utilization for grain production. There are some reports on N-management [6] and the optimization of population density per unit area [11] for the maximum harvest of maize. However, the relationship between maize forage yield and plant density is not well established. Maize producers require more information on how N-fertilization and plant density practices affect dry-matter yield and quality.

Keeping the above information in view, the present study was conducted to (1) determine the growth and growth efficiency of maize at different levels of N-fertilizer and plant population and (2) determine the optimum N-fertilizer application and plant population density for the maize growing farmers. We examined the plant height, light interception (LI), leaf area index (LAI), total dry-matter (TDM), grain yield (GY), and harvest index (HI) of maize plants in treated soils. In addition, soil-plant-analysis development (SPAD) values, dry-mass (DM), and harvest index (HI) of maize plants were also evaluated. We believe that this work would therefore aid in the development of production systems (seed rate, fertilizing) and the profitability of the crop in Bangladesh as well as maize growing countries in the world.

## 2. Materials and Methods

### 2.1. Experimental Site and Land Preparation

Field experiments were conducted during the *rabi *season (November–February) in the experimental field of the BSMR Agricultural University at Gazipur in Bangladesh. The experimental site is geographically situated at 23°N latitude and 91°E longitudes with an elevation of 6 m from sea level at the eastern part of Bangladesh. The soil of the experimental plot before application of farm yard manure and nitrogen was silty clay loam having pH 6.5, organic C content was moderate, 1.13%, total N (%) 0.08, available P (Olsen) 9 ppm, exchangeable K (meq 100 g^−1^ soil) 0.20, exchangeable Ca (meq 100 g^−1^ soil) 4.5, available S 14 ppm, Zn 10 ppm, and Fe was 370 ppm. The experimental field was cleared, and weeds were manually removed. The soil was ploughed and harrowed with power-tiller, leveled carefully to get a well-pulverized soil, and divided into plots. Each plot was surrounded by 20 to 25 cm high mud plastered levee to prevent the entering of irrigation water. Recommended practices for disease and insect control were followed.

### 2.2. Experimental Design and Fertilization of Crop

Field plots (2 × 4 m) were arranged in a split-plot factorial fitted to randomized complete block design (RCBD) with 3 replicates. Adjacent blocks were separated by a 2 m alley, and main plots were separated by a 1 m alley. Experimental treatments were repeated on the same 2 × 4 m plot area each year. The main-plot treatments were 3 plant populations (53000, 66000, and 80000 plants ha^−1^) and 4 rates of N (0, 100, 140, 180 and 220 kg N ha^−1^) as subplots. The treatment combinations for the experiment were shown in Table 1. Clean and healthy matured maize seeds (germination rate > 95%) were sown at the spacing of 75, 60, and 50 cm between rows and 25 cm within rows to give population density of 53,000, 66,000 and 80,000 plants ha^−1^. The variety of maize used for the experiment was *Z. mays* cv. “Mohor”. We chose “Mohor” because it is one of the popular maize varieties in Bangladesh.

The well-decomposed farmyard manure (FYM—a combination of animal manure, animal urine, and plant materials used for animal bedding and waste straw feed) at 10 t ha^−1^ was applied during the final land preparation. The land was fertilized (by hand) with 110 kg triple super phosphate (TSP), 50 kg muriate of potash (MP), 120 kg gypsum ha^−1^, respectively [12]. One third of the urea and the whole amount of TSP, MP and gypsum were applied at the time of final land preparation (i.e., 2 w prior to sowing), while the remaining urea was spitted equally and top-dressed at 28 and 53 days after sowing (DAS). Weeds were manually controlled and irrigation was done as and when necessary. Recommended practices for disease and insect control were followed as and when necessary.

### 2.3. Measurement of Plant Height

The height of five randomly selected plants was measured from the base of the plant to the tip of the tallest leaf. The height of each plant was recorded in cm, and the mean values of 5 plants for each plot were determined.

### 2.4. Determination of Leaf Area Index (LAI) and Light Interception (LI)

Leaf area index (LAI) was determined at 15-day intervals (35, 50, 65, and 80 DAS) by an automatic leaf area meter (Model AMM-8, Hayashi Denko Co. Ltd., Tokyo, Japan) using the following formula: LAI = total leaf area of a plant (m^2^)/ground area covered by a plant (m^2^), described by Pierce and Running [13].

Light interception (LI) by the crop community was recorded at five times, for example, 35, 50, 65, 80, and 95 DAS of maize by Sunfleck Ceptometer (Model Decagon, Pulman, Washington, USA) using the following formula: light transmission ratio (LTR) = (*I*/*I*_0_) × 100, where (*I*_0_) is photosynthetically active radiation (PAR) received on the top of the canopy and *I* is PAR at different depth of the canopy. 

### 2.5. Measurement of Crop Growth Rate (CGR) and Relative Growth Rate (RGR)

Crop growth rate (CGR) is in the increase in plant dry materials per unit area of land per unit time. CGR values were estimated at 15-day interval as described by Hunt [14]. The following formula was used: CGR (g^2^ m^−2^ day^−1^) = (*W*_2_ − *W*_1_)/(*T*_2_ − *T*_1_), where *W*_1_ is total dry weight at time *T*_1_ and *W*_2_ is the total dry weight at time *T*_2_.

Relative growth rate (RGR) is in the increase in plant materials per unit of materials present per unit time. RGR was determined using the following formula proposed byHunt [14]: RGR (g g^−1^ day^−1^) = (ln⁡*W*_1_ − ln⁡*W*_2_)/(*T*_1_ − *T*_2_), where *W*_1_ is total dry weight at time *T*_1_, *W*_2_ is total dry weight at time *T*_2_, and ln is natural logarithm.

### 2.6. Determination of Soil-Plant-Analysis Development (SPAD) Value

Leaf chlorophyll content may be used as an indirect indicator of crop N status. Chlorophyll meter values (SPAD) were taken using a portable SPAD meter (Model SPAD-502, Minolta crop, Ramsey, NJ) starting from 60 DAS with 15-day interval. The instrument measures transmission of red light at 650 nm, at which chlorophyll absorbs light, and transmission of infrared light at 940 nm, at which no absorption occurs. On the basis of these two transmission values, the instrument calculates a SPAD value that is well correlated with chlorophyll content [15].

### 2.7. Measurement of Grain Yield, Dry Matter and Harvest Index

After the final harvest, the cobs from each plot were separated, cleaned, dried, and weighed separately. Grain yield of each plot was recorded kg ha^−1^ individually and adjusted at 14% moisture content. Dry-mass (DM) of roots and shoots was determined after drying the samples at 72°C until the constant weight was attained.

The harvest index (HI) was computed as the ratio of grain yield (GY) to the total above ground DM yield. The following formula was used: HI (%) = (grain yield/total biological yield) × 100.

### 2.8. Chemical Analysis of Leaf

Leaves were collected and carried out in the laboratory of the Department of Soil Science, BSMRAU, Salna, Gazipur. Leaf N-content is an important physiological parameter that indicates the plant N status [16]. The total nitrogen concentration in maize leaves was determined by modified the Kjeldahl digestion colorimetric method as described by Cataldo et al. [17].

### 2.9. Statistical Analysis

All data collected were analyzed using analysis of variance (ANOVA) followed by protected Fisher's least-significant difference (LSD) test. The means were separated by the LSD at the *P* = 0.05 level of probability [18]. All the experiments were performed twice, and replicates (3) were averaged.

## 3. Results and Discussion

### 3.1. Plant Height

**Figure 1:**
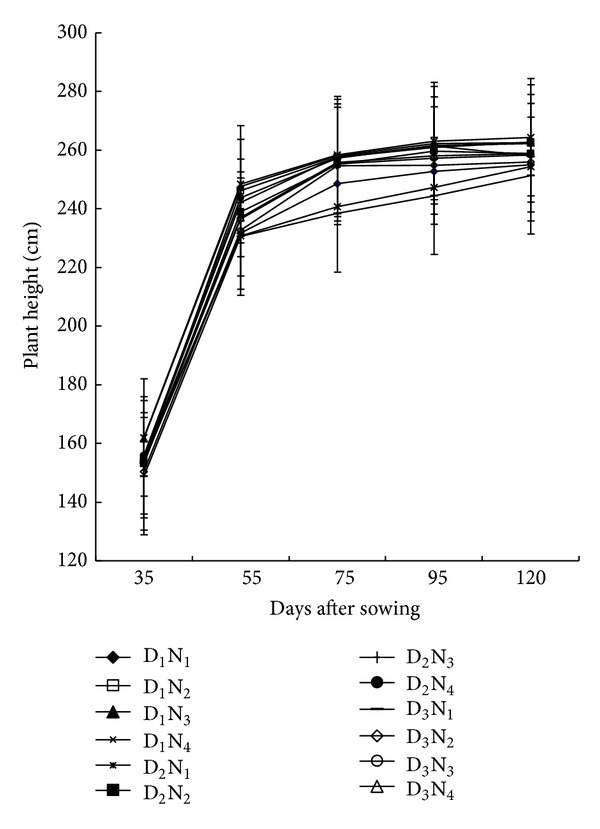
Plant height of maize at successive growth stages as affected by planting density and N levels (D_1_ = 53,000 plant/ha, D_2_ = 66,000 plant/ha, D_3_ = 80,000 plant/ha, N_1_ = 100 kg N/ha, N_2_ = 140 kg/ha, N_3_ = 180 kg N/ha, N_4_ = 220 kg N/ha. Bar showed ± SE value).

Figure 1 shows the plant height in response to different N levels and plant population over time. Plant height increased progressively over time attaining the highest at physiological maturity. The rate of increase, however, varied depending on the growth stages. A significant variation in plant height was observed due to planting density and N-rates except at 35 DAS. The influence of both planting density and N-rates was first apparent at 55 DAS, and the difference among them persisted throughout the growing period. Sparsely populated plants (53,000 ha^−1^) with 220 kg N ha^−1^ were the tallest and were statistically at par with that received 180 kg N ha^−1^.

This might be due to receiving sufficient amount of light and nutrients. On the contrary, densely populated plants with lower N-rates were shorter at all the growth stages. Our study supports the work of Pandey et al. [19] who observed that plant height of maize increased greatly when the seeds were planted sparsely and sufficient amount of N was applied. 

### 3.2. Leaf Area Index (LAI)

**Figure 2:**
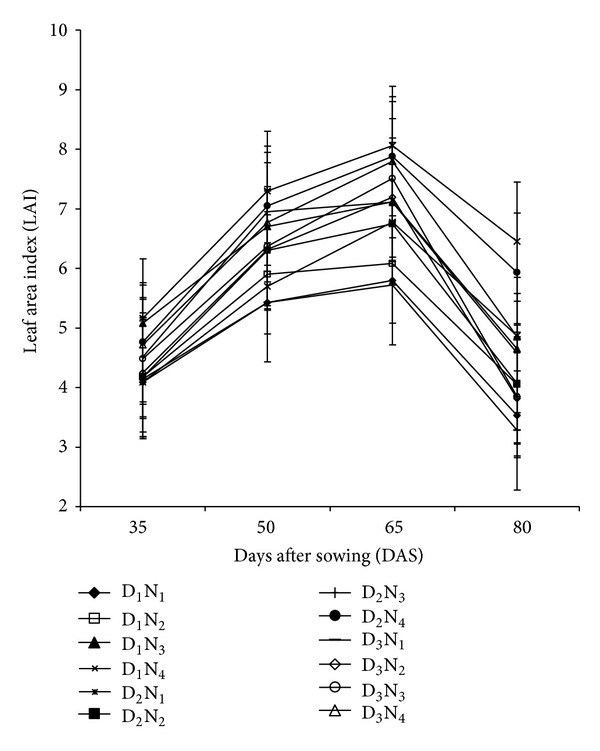
Leaf area index at different growth stages of maize as affected by planting density and N-fertilizer application (D_1_ = 53,000 plant/ha, D_2_ = 66,000 plant/ha, D_3_ = 80,000 plant/ha, N_1_ = 100 kg N/ha, N_2_ = 140 kg/ha, N_3_ = 180 kg N/ha, N_4_ = 220 kg N/ha. Bar showed ± SE value).

LAI of maize with different levels of N-fertilizer and planting density showed substantial differences over the growth stages (Figure 2). LAI increased sharply reaching peak at 65 DAS and then decreased irrespective of treatment differences. The rate of decrease of LAI after attaining peak was more rapid. LAI decreased at 65 DAS reflecting the loss of some existing leaves through senescence. Among the planting densities, 53,000 plants ha^−1^ showed higher LAI throughout the growing period; however, maximum LAI was obtained from 220 kg N ha^−1^ with 53,000 plants ha^−1^ irrespective of growth stages. The increased LAI might be due to the increased availability of N under the higher levels of N-fertilizer with lower population, which resulted in larger leaves. From this result, it is evident that increasing population density tended to depress LAI irrespective of N-levels. Reduction of LAI might be due to an interplant competition (for nutrient and space) within the community. Similar trend in reduction of LAI was also reported in maize and forage maize by Gardner et al. [20] and Okpara and Omaliko [21]. 

### 3.3. Light Interception (LI)

**Figure 3:**
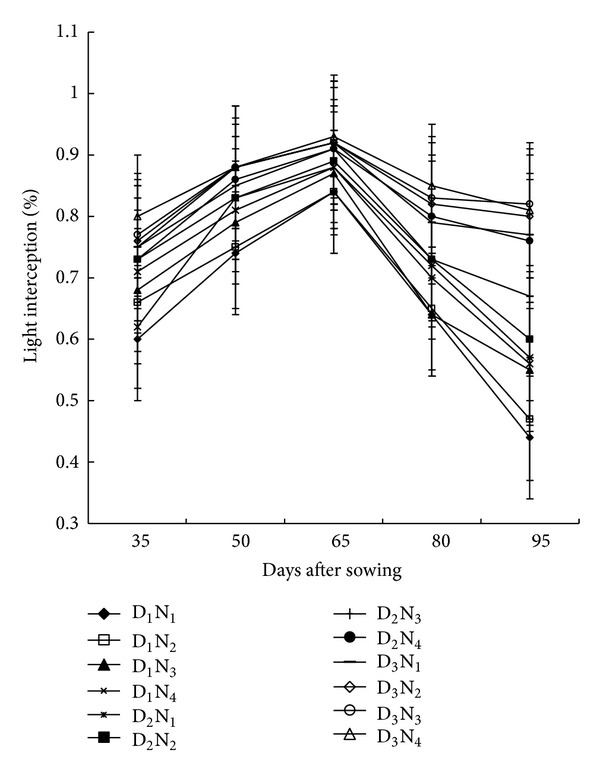
Light interception by maize canopy as affected by planting density and N-fertilizer application at different growth stages (D_1_ = 53,000 plant/ha, D_2_ = 66,000 plant/ha, D_3_ = 80,000 plant/ha, N_1_ = 100 kg N/ha, N_2_ = 140 kg/ha, N_3_ = 180 kg N/ha, N_4_ = 220 kg N/ha. Bar showed ± SE value).

 Planting density and N-fertilizer application differed substantially in LI. LI varied from 58 to 80% at 35 DAS and increased maximum at 65 DAS and thereafter it declined (Figure 3). Combination of D_3_N_4_ (80,000 plants ha^−1^ × 220 kg N ha^−1^) was favorable for light penetration to the upper of the canopy, which resulted better LI and the lowest LI was found in D_1_N_1_ (53,000 plants ha^−1^ × 100 kg N ha^−1^). Higher light absorption by the treatment D_3_N_4_ (80,000 plants ha^−1^ × 220 kg N ha^−1^) is presumably because of larger leaf surface availability for photosynthesis as evident by higher leaf dry-weight. Pepper [22] and Amanullah et al. [23] also reported that increased plant densities can promote utilization of solar radiation by maize canopies. On the contrary, it is reported that increased maize population/unit area decreased the availability of light into the maize canopy. This indicates that population is the main factor influencing the net radiation absorbed by the plants. Higher penetration of radiation below the canopy indicates lower interception of solar radiation at the canopy. The maximum light was intercepted at 65 DAS corresponded to higher LAI. The more the LAI, the greater the light interception. The LI was less at pretasseling and grain filling stages, which might be due to lower LAI in these stages of crop growth. However, efficiency of conversion of intercepted solar radiation into economic maize yields may decrease with high plant population density because of mutual shading of plants [24].

### 3.4. Crop Growth Rate (CGR)

**Figure 4:**
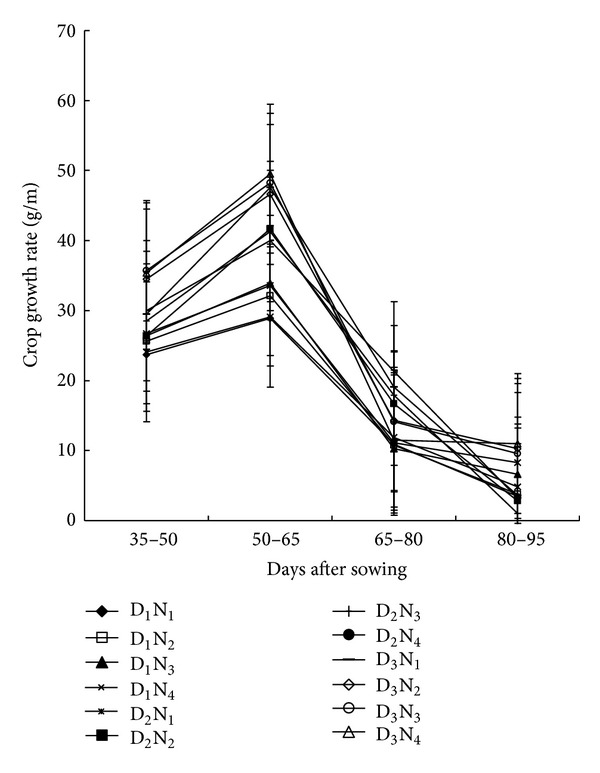
Crop growth rate of maize as affected by N-fertilizer and planting density at different growth stages (D_1_ = 53,000 plant/ha, D_2_ = 66,000 plant/ha, D_3_ = 80,000 plant/ha, N_1_ = 100 kg N/ha, N_2_ = 140 kg/ha, N_3_ = 180 kg N/ha, N_4_ = 220 kg N/ha. Bar showed ± SE value).

**Figure 5:**
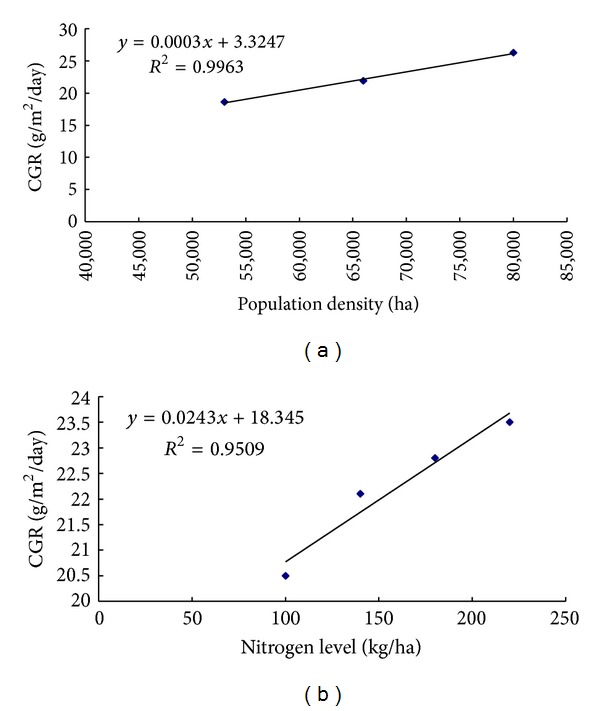
Functional relationship between (a) population density and CGR, (b) nitrogen level and CGR.

 Regardless of treatments, CGR increased progressively time reaching peak during 50 to 65 DAS and thereafter declined till maturity (Figure 4). Reduction in growth rate with plant age was probably due to cessation of vegetative growth, loss of leaves, and senescence. Similar reduction in the maize and in the late season of forage maize was also reported by Padmaja [25] and Okpara and Omaliko [21]. Treatment variation during the later growth periods was not significant though the differences were apparent in the initial 35 to 50 DAS up to 55 to 60 DAS. Among the N-levels, 220 kg N ha^−1^ showed maximum CGR throughout growth period followed by 180 kg N ha^−1^. The highest CGR was associated with the combination of D_3_N_4_ followed by D_3_N_3_, while the lowest CGR was in the plants at D_1_N_1_ treatment. In general, increase in planting density increased CGR in all the N-levels; CGR response to population density and N-rates followed a linear relationship suggesting that 97 and 99% of total variation in CGR could be accounted for by the linear function of N and population density, respectively (Figure 5). Therefore, the functional relationship suggests that higher the N-levels and population density, the greater was the CGR of maize plant.

### 3.5. Relative Growth Rate (RGR)

**Figure 6:**
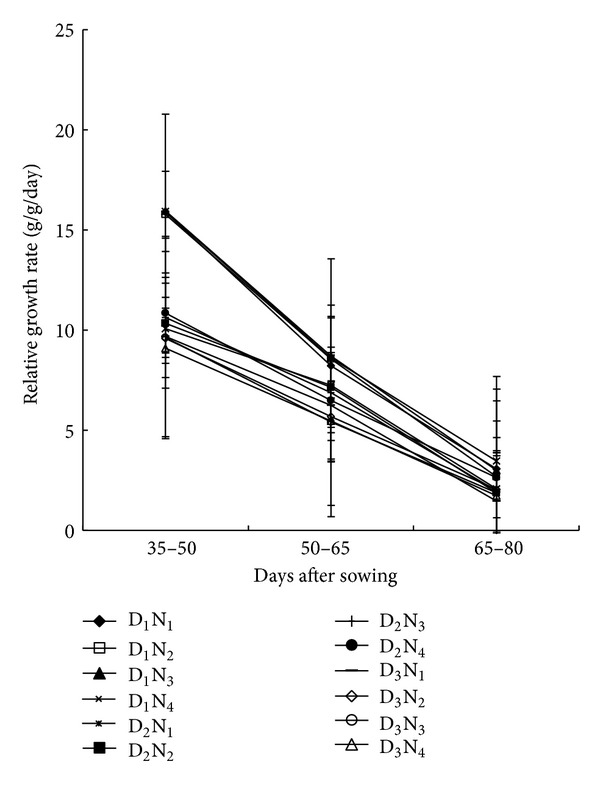
Relative growth rate of maize as affected by population density and N level at different growth period (D_1_ = 53,000 plant/ha, D_2_ = 66,000 plant/ha, D_3_ = 80,000 plant/ha, N_1_ = 100 kg N/ha, N_2_ = 140 kg/ha, N_3_ = 180 kg N/ha, N_4_ = 220 kg N/ha. Bar showed ± SE value).

 Irrespective of treatments, RGR was more at early stage (30 to 50 DAS) and showed a decreasing trend with the advancement of plant age (Figure 6). The decrease in RGR was probably due to the increase of metabolically active tissue, which contributed less to the plant growth. The decrease in RGR might be also due to the decrease in net assimilation rate (NAR). Variations in RGR across the treatments were not apparent in the later growth period, but the differences were observed in the early growth period. Sparsely populated plants with 100 kg N ha^−1^ (D_1_N_1_) gave the highest RGR, while the lowest PGR was found in densely populated plants (80,000 ha^−1^) with higher N-rates (220 kg ha^−1^). Similar results were reported for maize by Ahmad et al. [26] and for rice by Hussain et al. [27].

### 3.6. SPAD Values and N-Content in Maize Leaves

 Regardless of treatment, SPAD values decreased with the plant age (Table 2). Maximum SPAD values were observed at 60 DAS which declined progressively reaching the lowest at 105 DAS. Similar trend was also observed in the case of N-content in leaves. The higher SPAD values and N-content of maize leaves at 60 DAS were probably due to the less sink demand for N from the source (leaf). Conversely, lower SPAD values and N-content at 75 DAS and afterwards might have been due to remobilization of N from leaves to reproductive organs as grain formation was started after 60 DAS. Planting density exerted significant influence on SPAD values and N-content in leaves. However, higher values of both the parameters were obtained from sparsely populated plants (53,000 ha^−1^) at 60 DAS compared to other populating densities. Response of SPAD value and N-contents to N-rates was also found significant. SPAD value and N-content in the maize leaves increased as N-level increased. Planting density and N-rates interaction effects on SPAD values and N-content were also statistically significant. SPAD values and N-content in leaves increased with the increase of N-levels irrespective of population density.

The highest SPAD value and N-content were found in the plants treated with 220 kg N ha^−1^ regardless of planting density followed by 180 kg N ha^−1^. Our results corroborated the findings of Feibo et al. [28] and Zhao et al. [29] who reported that high correlations were recorded between SPAD readings, total leaf chlorophyll, and higher level of N-fertilizers application in short- season cotton (24.2 to 25.0 kg ha^−1^) and winter wheat (180 kg ha^−1^).

**Figure 7:**
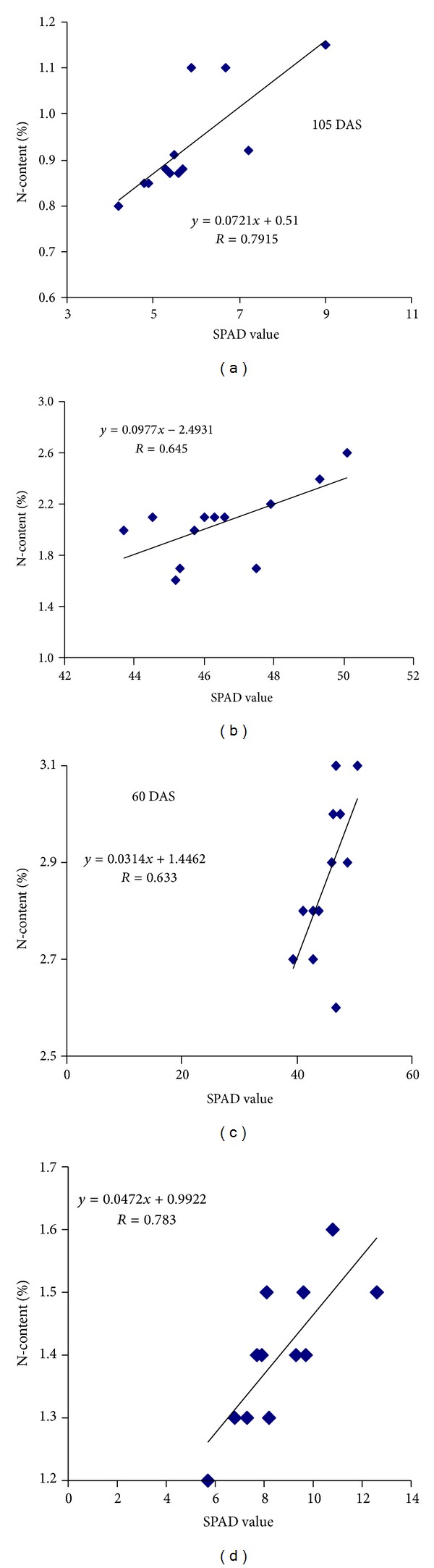
Relationship between SPAD values and N-content at different growth stages of maize plants.

The relationship between SPAD values and N-content is linearly associated at different growth stages (Figure 7), suggesting that as SPAD values were increased, N-content in leaves was also increased linearly. However, the functional relationship indicates that over 50 to 62% N-content variation in maize leaves can be attributed to the difference in SPAD values. 

### 3.7. Total Dry Matter Production

Total dry matter production (TDM) increased progressively with the progressive increase in planting densities and N-levels. Densely populated plants (80,000 ha^−1^) had accumulated more DM than the sparsely (53,000 ha^−1^) planted ones (Table 3). Plants (80,000) grown with 220 kg N ha^−1^ produced maximum TDM followed by 140 and 180 kg N ha^−1^. This might be due to higher number of plants per unit area. The DM production was largely a function of photosynthetic surface, which was favorably influenced by N-fertilization. The increase (60%) in DM production of cotton was due to N-fertilization also reported by Oosterhuis et al. [30] and Dubeux et al. [31]. Our results were compared favorably with those of Lucus and Remison [32] who observed linear trends of dry matter production in maize to increasing population density.

### 3.8. Interrelationships among the Plant Characters

Table 4 shows the correlation between different plant characters of maize. GY had significant positive correlation with total biomass produced and LAI. LTR was found negatively correlated with grain yield. HI has correlated with SPAD value, N-content in leaf, and grain yield.

TDM was significantly associated with LTR and LAI. Contribution of leaf area and LTR for variation in TDM accumulation indicated that leaf area was the most influential factor for determining TDM than the other parameters. LAI was positively correlated with N-content in leaf and LTR. N-content in the leaf had significant positive influence on SPAD value.

## 4. Conclusion

The results of this study conclusively reveal that the plant height was higher in sparsely populated plants with varying doses of N. Plants grown with 180 kg N ha^−1^ with 80,000 plants ha^−1^ had larger foliage, greater light absorption, and growth efficiency that eventually resulted in higher grain yield. On the basis of the obtained results, we concluded that growth efficiency could substantially be increased by adjusting population density with sufficient amount of N-fertilizer. Further experiments should be conducted considering different combinations of major/minor nutrient elements to fully exploit the growth parameters of maize crop under local conditions. 

## Figures and Tables

**Table 1:** The treatment combinations used in this study.

Treatment combination	Plants ha^−1^	Plant spacing (cm)	Kg N ha^−1^
D_1_N_1_	53,000	75 × 25	100
D_1_N_2_	53,000	75 × 25	140
D_1_N_3_	53,000	75 × 25	180
D_1_N_4_	53,000	75 × 25	220

D_2_N_1_	66,000	60 × 25	100
D_2_N_2_	66,000	60 × 25	140
D_2_N_3_	66,000	60 × 25	180
D_2_N_4_	66,000	60 × 25	220

D_3_N_1_	80,000	50 × 25	100
D_3_N_2_	80,000	50 × 25	140
D_3_N_3_	80,000	50 × 25	180
D_3_N_4_	80,000	50 × 25	220

**Table 2:** Effect of N-fertilizer and plant density on SPAD value and N-content in maize leaf at different growth stages.

Interaction of population and N-fertilizer (000' No./ha × kg/ha)	60 DAS		75 DAS		90 DAS		105 DAS	
	SPAD value	% N-content	SPAD value	% N-content	SPAD value	% N-content	SPAD value	% N-content
53 × 100	46.8	2.9	44.5	2.1	8.2	1.3	5.6	0.87
53 × 140	48.7	2.9	46.0	2.1	9.3	1.4	5.7	0.88
53 × 180	47.6	3.0	47.9	2.2	9.6	1.5	7.2	0.92
53 × 220	50.5	3.1	49.3	2.4	12.6	1.5	9.0	1.15
66 × 100	42.9	2.7	45.3	1.7	7.3	1.3	4.9	0.85
66 × 140	43.7	2.8	45.7	2.0	7.9	1.4	5.4	0.87
66 × 180	46.0	2.9	46.6	2.1	8.1	1.5	5.5	0.91
66 × 220	46.7	3.1	49.0	2.6	10.8	1.6	6.7	1.12
80 × 100	39.3	2.7	45.2	1.6	5.7	1.2	4.2	0.8
80 × 140	41.2	2.8	43.7	2.0	6.8	1.3	4.8	0.85
80 × 180	42.9	2.8	46.3	2.1	7.7	1.4	5.3	0.88
80 × 220	46.3	3.0	47.5	1.7	9.7	1.4	5.9	1.1
LSD (0.05)	1.72	NS	NS	0.15	0.41	0.16	0.99	0.06
CV (%)	3.2	4.6	2.2	5.4	2.7	5.1	9.9	4.7

DAS: days after sowing; SPAD: soil-plant-analysis development; NS: not significant; LSD: least significant difference; CV: coefficient of variation.

**Table 3:** Interaction effect of population density and N-fertilizer rates on yield and yield parameters of maize.

Interaction of population and N-fertilizer (000' No./ha × kg/ha)	Grain yield (t/ha)	Stover yield (t/ha)	TDM (t/ha)	HI (%)
53 × 100	2.52	9.18	11.19	21.51
53 × 140	3.92	8.76	12.69	27.91
53 × 180	4.43	9.0	13.43	29.98
53 × 220	4.40	9.83	13.87	27.92
66 × 100	3.20	11.03	14.23	22.48
66 × 140	4.31	11.17	15.48	27.84
66 × 18	4.70	11.01	15.71	29.91
66 × 220	4.58	12.06	16.64	27.17
80 × 100	3.50	12.42	15.92	21.98
80 × 140	4.67	13.59	18.26	25.57
80 × 180	5.03	13.59	18.62	27.01
80 × 220	4.62	15.17	19.79	23.34
LSD (0.05)	0.3	0.86	0.86	1.49
CV (%)	9.4	6.39	6.39	5.83

TDM: total dry matter; HI: harvest index; stover yield means dry-mass of roots and shoots.

**Table 4:** Interrelationship among the different plant characters of maize plant.

Characters	N-content in leaves (%)	LTR	LAI	TDM	GY	HI
SPAD value	0.885**	0.273^NS^	0.503^NS^	−0.253^NS^	0.252^NS^	0.621*
N-content in leaves (%)	—	−0.009^NS^	0.709*	−0.006^NS^	0.495^NS^	0.649*
LTR	—	—	−0.597*	−0.963**	−0.653*	0.189^NS^
LAI	—	—	—	0.605*	0.722*	0.292^NS^
TDM	—	—	—	—	0.713*	0.177^NS^
GY	—	—	—	—	—	0.613*

LTR: light transmission ratio; LAI: leaf area index; TDM: total dry-matter; GY: grain yield; HI: harvest index; SPAD: soil-plant-analysis development; NS: not significant; *significant at 5% level; **significant at 1% level.

## References

[1] USDA and FAS Grain Zea production maps and statistics. http://www.fas.usda.gov/psdonline/.

[2] Karim R (1992). *Studies on Maize in Bangladesh*.

[3] BBS (1995). *Statistical Pocketbook of Bangladesh*.

[4] Agricorner World top ten corn producers 2009. http://www.agricorner.com/world-top-ten-corn-producers-2009/.

[5] Lomte MH, Khuspe VS (1987). Effect of plant densities, P levels and antitranspirants on the yield of summer groundnut. *Journal of Maharashtra Agricultural Universities*.

[6] Sanjeev K, Bangarwa AS (1997). Yield and yield components of winter maize (*Zea mays* L.) as influenced by plant density and nitrogen levels. *Agricultural Science Digest*.

[7] Natr L (1992). Mineral nutrients an iniquitous stress factor for photosynthesis. *Photosynthetica*.

[8] Khot RB, Umrani NK (1992). Seed yield and quality parameters of “African Tall” maize as influenced by spacing and level of nitrogen. *Indian Journal Of Agronomy *.

[9] Hardas G, Karagianne-Hrestou M (1985). Long-term fertilizer trail in the Kopais area with a 2-year rotation of maize and wheat. The effect of NPK application on yield. *Field Crops Research*.

[10] Singh DP, Rana NS, Singh RP (2000). Growth and yield of winter maize (*Zea mays* L.) influenced by intercrops and nitrogen application. *Indian Journal of Agronomy*.

[11] Sabir MR, Shah SAH, Shahzad MA, Ahmed I (2001). Effect of plant population on yield and yield components of maize. *Journal of Agricultural Research*.

[12] BARI (2006). *Handbook of Agricultural Technologies (Krishi Projukti Hatboi)*.

[13] Pierce LL, Running SW (1988). Rapid estimation of coniferous forest leaf area index using a portable integrating radiometer. *Ecology*.

[14] Hunt R, Rose DA, Edwards DAC (1978). The fitted curve in plant growth studies. *Plant Physiology*.

[15] Markwell J, Osterman JC, Mitchell JL (1995). Calibration of the Minolta SPAD-502 leaf chlorophyll meter. *Photosynthesis Research*.

[16] Lemaire G, Jeuffroy M-H, Gastal F (2008). Diagnosis tool for plant and crop N status in vegetative stage. Theory and practices for crop N management. *European Journal of Agronomy*.

[17] Cataldo DA, Schrader LE, Youngs BL (1974). Analysis by digest and colorimetric assay of total nitrogen in plant tissue high in nitrate. *Crop Science*.

[18] Gomez KA, Gomez AA (1984). *Statistical Procedure For Agricultural Research*.

[19] Pandey AK, Prakesh V, Mani VP, Singh RD (2000). Effect of rate of nitrogen and time of application on yield and economics of baby corn. *Indian Journal of Agronomy*.

[20] Gardner PF, Pearce RB, Mitehell RL (1985). *Physiology of Crop Plants*.

[21] Okpara DA, Omaliko GP (1999). Influence of plant density and nitrogen fertilization on late-season forage maize. *Journal of Applied Chemistry and Agricultural Research*.

[22] Pepper GE (1974). *The effect of leaf orientation and plant density on the yield of maize (Zea mays L.) [Ph.D. thesis]*.

[23] Amanullah A, Asif M, Nawab K (2010). Impact of planting density and P-fertilizer source on the growth analysis of maize. *Pakistan Journal of Botany*.

[24] Buren LL (1970). *Plant characteristics associated with barrenness in maize [Ph.D. thesis]*.

[25] Padmaja M, Sreelatha D, Rao KL (1999). Effect of nitrogen on nutrient uptake in maize (*Zea mays* L.) types. *Journal of Research ANGRAU*.

[26] Ahmad R, Mahmood A, Ikraam M, Hassan B (2002). Irrigation methods and maize productivity. *International Journal of Agriculture and Biology *.

[27] Hussain ASMI, Haque MA, Hossain D, Hassan MZ, Bari N (2011). Effect of population density and nitrogen fertilizer on growth and yield of BRRI dhan29. *Bangladesh Research Publications Journal*.

[28] Feibo W, Lianghuan W, Fuhua X (1998). Chlorophyll meter to predict nitrogen sidedress requirements for short-season cotton (*Gossypium hirsutum* L.). *Field Crops Research*.

[29] Zhao D, Reddy KR, Kakani VG, Reddy VR (2005). Nitrogen deficiency effects on plant growth, leaf photosynthesis, and hyperspectral reflectance properties of sorghum. *European Journal of Agronomy*.

[30] Oosterhuis DM, Chipamaunga J, bate GC (1983). Nitrogen uptake of field-grown cotton. I. distribution in plant components in relation to fertilization and yield. *Experimental Agriculture*.

[31] Dubeux JCB, dos Santos MVF, de Andrade Lira M (2006). Productivity of *Opuntia ficus-indica* (L.) Miller under different N and P fertilization and plant population in north-east Brazil. *Journal of Arid Environments*.

[32] Lucus EO, Remison SU (1984). Effect of population density on yield and dry-matter partition of varieties in Nigeria. *The Indian Journal of Agricultural Sciences*.

